# Defect- and Interface-Induced Dielectric Loss in ZnFe_2_O_4_/ZnO/C Electromagnetic Wave Absorber

**DOI:** 10.3390/nano12162871

**Published:** 2022-08-20

**Authors:** Hao Shen, Zhen Wang, Chun Wang, Pengfei Zou, Zhaoyang Hou, Chunlong Xu, Hongjing Wu

**Affiliations:** 1 Department of Applied Physics, School of Science, Chang’an University, Xi’an 710064, China; 2 School of Science, Xi’an Shiyou University, Xi’an 710064, China; 3 MOE Key Laboratory of Material Physics and Chemistry Under Extraordinary Conditions, School of Physical Science and Technology, Northwestern Polytechnical University, Xi’an 710072, China

**Keywords:** ZnFe_2_O_4_/ZnO/C, defects and interfaces, dielectric loss, electromagnetic wave absorption

## Abstract

Controlling defects and interfaces in composite absorbers can effectively regulate electromagnetic (EM) parameters and enhance the electromagnetic wave (EMW) absorption ability, but the mechanism still needs to be further elucidated. In this study, ZnFe_2_O_4_/ZnO/C composite was synthesized via the hydrothermal method followed by post-annealing in different atmospheres. Defects and interfaces were characterized by Raman, PL spectroscopy, XPS and TEM, and their relationship with dielectric loss and EMW absorption performance was discussed in detail. Results show that the N_2_-annealed ZnFe_2_O_4_/ZnO/C composite with abundant defects and interfaces as well as an optimized composition exhibits excellent EMW dissipation ability, with a *RL_min_* value of −17.4 dB and an *f_e_* of 3.85 GHz at a thickness of 2.28 mm. The excellent EMW absorption performance originates from suitable impedance matching, significant conduction loss and strong dielectric loss (interfacial polarization, diploe polarization and defect polarization) dominated by lattice defects and interfaces. This study provides a view into the relationship between defects, interfaces, EM parameters and EMW absorption ability, and also suggests an effective way to promote EMW dissipation ability of the absorbers by controlling defects and interfaces.

## 1. Introduction

Nowadays, electromagnetic waves (EMW) pollution caused by the emerging 5G networks and widespread use of electronics devices is gradually causing potential negative impacts on precision electronic instruments [1], the ecological environment [2] and even human health [3,4,5]. EMW absorbing materials, which can absorb and dissipate electromagnetic (EM) energy, are widely believed to be able to effectively eliminate the hazards of EM pollution [6,7,8]. Great efforts have been made to find high-performance EMW absorbers with thin thickness and light weight. So far, numerous candidates have been extensively explored, including dielectric loss absorbers (such as carbon materials and conductive polymers) [9,10,11], magnetic loss materials (such as magnetic ferrites) [12,13,14], and dual-loss materials compose of dielectric loss materials and magnetic loss materials [15,16,17,18]. Among these absorbers, zinc ferrite (ZnFe_2_O_4_) has drawn dramatic attention due to the advantages of low-cost, corrosion resistance, and high magnetic loss. However, its poor dielectric loss ability and the bad impedance matching between permittivity and permeability hinder its application prospects.

Coupling ZnFe_2_O_4_ with dielectric materials (such as Fe_2_O_3_ [19], MnO_2_ [20], ZnO [21] and so on) is an effective strategy to improve the dielectric loss ability and obtain a better impedance matching. Among these dielectric materials, ZnO is regarded as a promising candidate due to its unique electronic property, chemical stability and tailored dielectric loss. It has been widely reported that the combination of ZnO with ferrites can effectively adjust the permittivity and improve their impedance matching. However, both ZnFe_2_O_4_ and ZnO materials suffer from poor electrical conductivity, and the introduction of carbon materials can effectively address this problem [22,23,24]. So far, various carbon materials have been used to prepare ZnFe_2_O_4_-based EMW absorbers, such as ZnFe_2_O_4_/carbon [25,26,27], ZnFe_2_O_4_/MWCNTs [28], ZnFe_2_O_4_/polypyrrole [29], ZnFe_2_O_4_/ZnO/rGO [21] and so on. For these composites, excellent EMW absorption ability can be achieved, due to the significant increase in complex permittivity, dielectric loss, conduction loss and good impedance matching.

However, when it comes to the reason for the enhancement of permittivity and dielectric loss, the most common explanation is the existence of the defects and the forming of interfaces between different components in the composites. These defects and interfaces can serve as polarization center, and leads to polarization and relaxation under alternating electromagnetic field, which cause the dielectric loss and consume the energy of incident EMW [7]. In most cases, defects in spinel structure (mainly Fe^2+^ in ZnFe_2_O_4_) and oxygen vacancy (V_O_) are the two types of defects considered and analyzed. Apart from them, other types of lattice defects also exist both in ZnFe_2_O_4_ and the dielectric loss components of these ZnFe_2_O_4_-based materials, which can also accumulate charges and form diploes with different dipole moments. However, their contribution to dielectric loss still lacks attention and investigation. Furthermore, the relationship between lattice defects, interfaces, complex permittivity, and dielectric loss ability remains unclear, resulting in a lack of theoretical basis for effectively controlling defects in materials to adjust complex permittivity and promote the EMW absorption ability.

Generally, various defects will be generated in materials during the material synthesis processes, which can be affected by numerous factors such as reaction temperature and time, annealing temperature and atmosphere. By optimizing these factors, defects can be well controlled to a certain extent [30]. Among all the strategies, post-annealing treatment is a widely used method, which can effectively control the defect as well as the interfaces and the content of compositions in composites materials, especially when the materials are post-annealed under different atmospheres. Therefore, it provides a feasible way to control defects and interfaces for the purpose of exploring their relationship with permittivity and EM wave absorption performance. 

In this work, ZnFe_2_O_4_/ZnO/C composites were synthesized via the hydrothermal method. The post-annealing process was carried out in different atmospheres to adjust the composition content and the contents of defects and interfaces. The EM parameters and EMW absorption performance of the composites were measured, and the relationship between defects, interfaces, permittivity and the dielectric loss ability was further explored. The results of this work will help to promote the EM wave absorption performance of absorbers by controlling the defects and interfaces.

## 2. Materials and Methods

### 2.1. Synthesis of ZnFe_2_O_4_/ZnO/C Samples

The ZnFe_2_O_4_/ZnO/C samples were synthesized via hydrothermal method, and the synthesis process is described in detail as follows. Firstly, 2 mmol of Zn(NO_3_)_2_, 2 mmol of Fe(NO_3_)_3_ and 8 mmol of D-glucose were dissolved in 60 mL deionized water. After stirring for 30 min, the obtained homogeneous solution was transferred to a 100 mL stainless-steel autoclave. The autoclave was heated to 180 °C in a muffle oven for 24 h. After that, the products were collected and washed three times each with ionized water and alcohol. After drying in an oven overnight, the ZnFe_2_O_4_/ZnO/C composite was obtained and marked as S1. The ZnFe_2_O_4_/ZnO/C composite (S1) was further post-annealed in air and N_2_ for 2 h at 550 °C, respectively, and the obtained samples were marked as S2 and S3.

### 2.2. Characterization 

The phase, structure and morphology of the samples were characterized by X-ray diffraction (XRD, PANalytical X’pert MPD PRO, Cu Kα, λ = 0.15406 nm, Malvern Panalytical, Malvern, UK), Raman spectra (WITec Alpha 300R, WITec, Ulm, Germany), field emission scanning electron microscopy (SEM, ZEISS GeminiSEM 300, ZEISS, Beijing, China) and Transmission Electron Microscope (TEM, Talos F200X, Thermo Fisher, Shanghai, China). Defects and chemical states were characterized by UV–Vis diffuse reflectance spectra (DRS, PE Lambda 950, Perkin Elmer, Shanghai, China), photoluminescence spectra (PL, Gangdong F-320, Gangdong, Tianjin, China) and X-ray photoelectron spectroscopy (XPS, Kratos Axis Ultra DLD, Kratos, Manchester, UK). The prepared samples were uniformly mixed with paraffin at a weight ratio of 1:1, and then pressed into a ring-shaped mold (d_out_ = 7.0 mm, d_in_ = 3.04 mm). The electromagnetic parameters were measured on an Agilent N5230A vector network analyzer (Agilent, Beijing, China) in frequency range of 2–18 GHz. 

## 3. Results and Discussion

**Figure 1:**
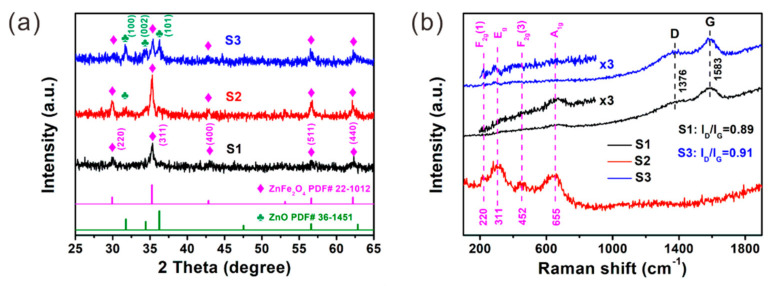
(**a**) XRD patterns and (**b**) Raman spectra of S1–S3.

The phase and crystal structure of the samples were investigated by XRD patterns (Figure 1a). For the as-prepared sample (S1), the diffraction peaks at 29.9°, 35.3°, 46.3°, 56.7° and 62.4° can be well assigned to the (220), (311), (400), (511), and (440) planes of the cubic spinel structure of ZnFe_2_O_4_ crystal (PDF# 22-1012). These peaks can also be clearly observed in the XRD spectra of S2 and S3. The full width at half maximum (FWHM) of the strongest (311) peak is 0.336°, 0.272° and 0.299° for S1–S3, respectively. The narrowing of this peak indicates the increase in crystal grains after the post-annealing process. The average crystallite size (*D*) of ZnFe_2_O_4_ grains calculated by using the Scherrer formula is 28 nm, 35 nm and 32 nm for S1–S3, respectively. Besides the typical peaks of ZnFe_2_O_4_, additional peaks related to the hexagonal wurtzite ZnO (PDF# 36-1451) can also be observed in the spectra of S2 and S3, which confirms the coexistence of ZnO and ZnFe_2_O_4_ in S2 and S3. For S2, the diffraction peak intensity of ZnO is much weaker than the (311) peak of ZnFe_2_O_4_, indicating the low content of ZnO in S2. The diffraction peaks of ZnO become much stronger in S3, while the peak of ZnFe_2_O_4_ becomes weaker, which indicates that compared with S2, S3 has a larger ZnO content and a lower ZnFe_2_O_4_ content.

The phase composition of the samples was further studied by Raman spectra, as displayed in Figure 1b. In the Raman spectrum of S2, several distinguishable peaks appear at 220 cm^−1^, 311 cm^−1^, 452 cm^−1^, and 655 cm^−1^, which are assigned to F_2g_(1), E_g_, F_2g_(3) and A_1g_ modes of ZnFe_2_O_4_ [31]. These peaks are significantly weakened or even disappeared in the spectra of S1 and S3, indicating the degradation of the ZnFe_2_O_4_ crystallinity, which is consistent with the results of XRD. Moreover, two additional peaks appear at ~1376 cm^−1^ and ~1583 cm^−1^, which are typical peaks for carbonaceous materials and are called as D band and G band, respectively [32]. The latter one is attributed to the stretching vibrations of the sp^2^ bonded carbon atoms, while the former one arises from the in-plane vibrations of disordered amorphous carbon, reflecting the defects and disorder within the structure of the carbonaceous materials. The intensity ratio of I_D_/I_G_ is usually used to evaluate the graphitization degree of the carbonaceous materials, and a higher ratio means more defects or a higher disorder degree [33]. The I_D_/I_G_ ratio is calculated to be 0.89 and 0.91 for S1 and S3 (Table 1), respectively. The large values indicate the high disorder degree of carbon component in these two samples. 

**Figure 2:**
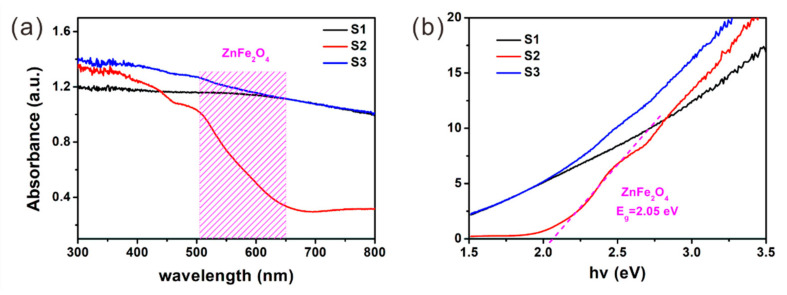
(**a**) UV-Vis diffuse reflectance spectra and (**b**) Tauc plot of S1–S3.

**Figure 3:**
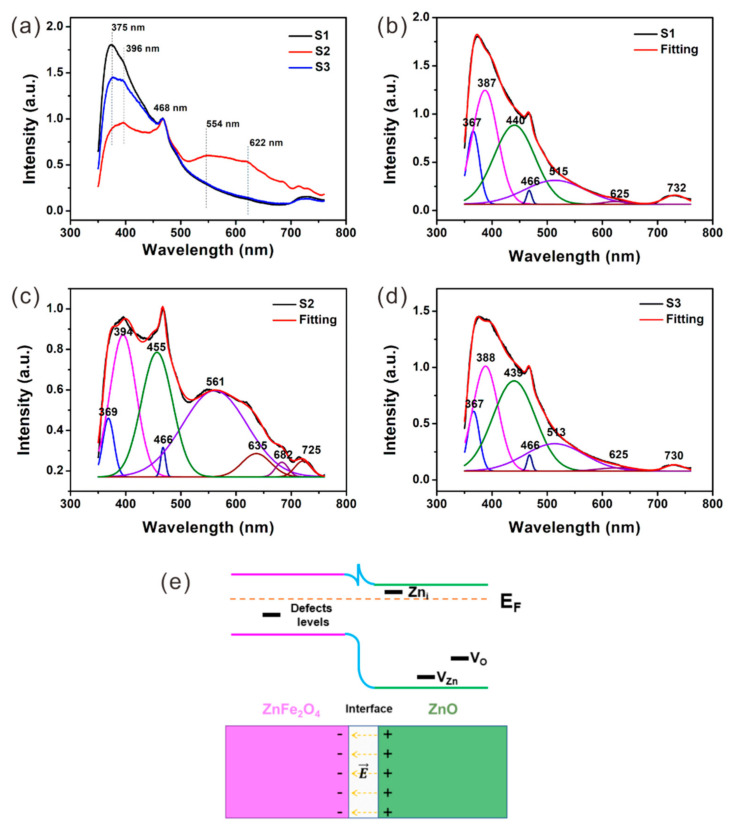
(**a**) PL spectra of S1–S3; Gaussian fitting of PL spectrum of (**b**) S1, (**c**) S2 and (**d**) S3; (**e**) energy band diagram and schematic diagram of ZnFe_2_O_4_/ZnO interface.

Lattice defects exist not only in the carbon component, but also in the crystal grains. In order to study the defects as well as the band structure of the crystals, UV-Vis diffuse reflectance spectra (DRS) and PL spectra were recorded and are displayed in Figure 2a and Figure 3a, respectively. Seen from the spectrum of S2 in Figure 2a, a distinct absorption edge related to ZnFe_2_O_4_ can be observed in range from ~500 to ~650 nm. The bandgap (Eg) of ZnFe_2_O_4_ is further calculated to be ~2.05 eV from the Tauc plot in Figure 2b. The corresponding near band-edge emission of ZnFe_2_O_4_ emits a distinguishable peak at ~622 nm, which can be detected in the PL spectra of all samples (Figure 3a). Moreover, several additional peaks and shoulders could also be observed in the PL spectra of the samples. Among which, the peak at 466 nm is caused by the instrument, which remains stable and can be used to calibrate the spectrum. Considering the bandgap of ZnFe_2_O_4_ (~2.05 eV) and ZnO (~3.36 eV), it is easy to conclude that the peaks at ~375 nm, ~394 nm and ~554 nm should be due to the band-to-band and defects-related emissions of ZnO, and the appearance of these peaks confirms that ZnO is present in all samples, including S1. 

For a better understanding of the defects-related emissions, Gaussian fitting of the spectra were conducted and displayed in Figure 3b–d. In terms of S1 (Figure 3b), the deconvoluted peaks at ~367 nm and ~387 nm is due to the band-to-band emission and near band-edge emission of ZnO, respectively. As reported by previous studies [34,35,36], the peak at ~440 nm is attributed to the transition between the Zn_i_ level (located ~0.22 eV below the conduction band) to the V_Zn_ level (located ~0.3 eV above the valance band). The emission peak at ~515 nm is widely recognized to be related to the electron–hole recombination between the conduction band and the V_O_ level [37,38]. As analyzed above, the peak at ~625 nm should be the near band-edge emission of ZnFe_2_O_4_ [39]. The last peak at ~732 nm may be generated by the deep-level defects in ZnO or ZnFe_2_O_4_. The Gaussian fitting result for S3 (Figure 3d) is similar to that of S1, with all the deconvoluted peaks appearing at similar positions. These deconvoluted peaks can also be obtained after a Gaussian fitting process to the spectrum of S2 (Figure 3c). Unlike S1 and S3, the V_O_-related emission of S2 is red-shifted to ~561 nm, which is due to the fact that the electron transitions occur between the Zn_i_ level and the V_O_ level rather than the conduction band and the V_O_ level [40]. Moreover, S2 has one extra peak at ~682 nm, which should be due to the deep level defects in ZnFe_2_O_4_, confirming the existence of lattice defects in ZnFe_2_O_4_ crystals.

Moreover, the ratio of the integrated intensity of the green peak (related to Zn_i_ and V_Zn_ defects) to the sum of the integrated intensities of the blue and purple peaks (related to the band structure) was calculated to be 0.89, 0.91 and 1.18 for S1–S3 (Table 1), respectively. The larger ratio of S3 indicates a higher defect content of Zn_i_ and V_Zn_ in S3, which is consistent with previous literature results that annealing in nitrogen favors the formation of Zn_i_ and V_Zn_ in ZnO materials [41]. Since the present of carbon in the samples can significantly absorb the visible light in the range of 500–750 nm (See Figure 2a), the emissions in this range are absorbed by the carbon component in the samples, resulting in the weak PL intensities in the spectra of S1 and S3. The carbon can be removed by air annealing, thus the emissions in this range can be clearly observed for S2, which make it seem that S2 has more V_O_ defects (strong purple deconvoluted peak at ~561 nm) than S1 and S2. The accurate content of V_O_ defects can be calculated by XPS, which will be discussed in the XPS section later.

Based on the above analysis, the energy band diagram of the ZnFe_2_O_4_/ZnO interface was drawn and is displayed in Figure 3e. Due to the different work function of ZnFe_2_O_4_ (~5.3 eV) [39] and ZnO (~ 4.5 eV) [42], electrons will diffuse from ZnO to ZnFe_2_O_4_, resulting in a built-in electric field in the depletion region. Electrons and holes are separated and accumulated at the different ends of the depletion region, as shown by the schematic diagram in Figure 3e. As analyzed above, various defects exist in both ZnFe_2_O_4_ and ZnO, and their energy levels are also marked in the energy band structure. The existence of interfaces and defects will accumulate electrons and form dipoles, which will periodically polarize and relax in an alternating electromagnetic field. Thus, it can be predicted that multiple defects-related and interface-related polarization and relaxation processes will occur when the samples are exposed to EM waves, which will certainly consume the absorbed EMW energy, leading to the attenuation of incident EM waves.

**Figure 4:**
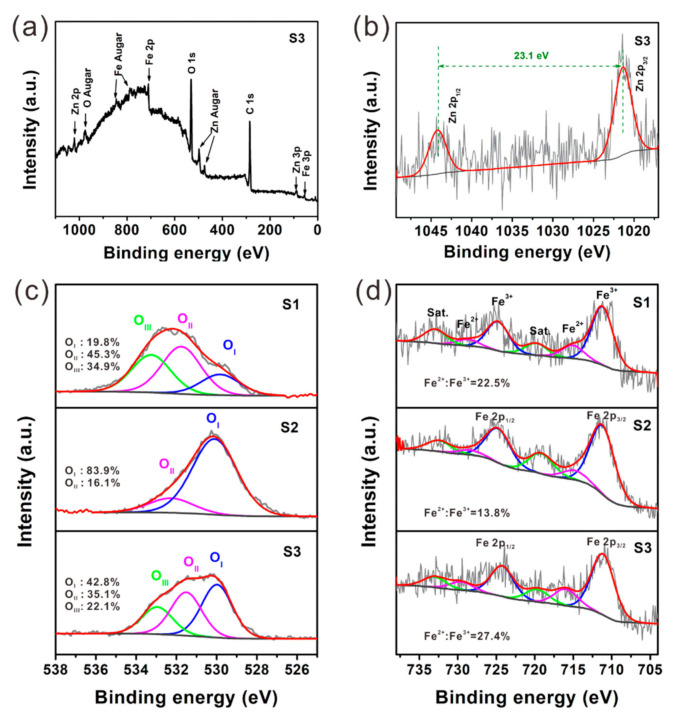
(**a**) Survey spectra and (**b**) Zn 2p spectra of S3; (**c**) O 1s spectra and (**d**) Fe 2p spectra of S1–S3.

The defects in spinel structure and V_O_ defects were further studied by XPS spectra. As shown in Figure 4a, all the distinguishable peaks can be well assigned to C, O, Fe, and Zn elements. The Zn 2p core-level spectra of S3 (Figure 4b) presents two peaks at 1021.2 eV and 1044.3 eV with an energy difference of 23.1 eV, which correspond to Zn 2p_3/2_ and Zn 2p_1/2_, respectively [40]. The O 1s spectra of S1can be deconvoluted into three components (Figure 4c), i.e., O_I_ (529.8 eV), O_II_ (531.7 eV) and O_III_ (533.2 eV), which are related to lattice oxygen, oxygen vacancies and the oxygen-containing functional group in the carbon or absorbed water, respectively [43]. The carbon and absorbed water can be removed after annealing in air, and thus, the O_III_ peak cannot be observed in the spectrum of S2. Generally, the air-annealing process benefits the growth of crystals and reduces V_O_ defects in oxides. As a result, the O_II_ component representing V_O_ defect shows a remarkable decrease, while the O_I_ peak corresponding to lattice oxygen becomes much stronger in the spectrum of S2. Compared with S1, S3 has a higher integrated intensity of O_I_ components, indicating that the crystallinity is improved after post-annealing in N_2_; while the integrated intensity of O_II_ and O_III_ components decreases, which reveals that post-annealing in N_2_ can help to remove oxygen vacancies and the oxygen atoms in carbon or absorbed water. As displayed in Figure 4d, several peaks related to Fe 2p_1/2_, Fe 2p_3/2_ and their satellites can be observed in the Fe 2p core-level spectra of S1–S3. Both Fe 2p_1/2_ and Fe 2p_3/2_ peaks can be further deconvoluted into two components, which are attributed to Fe^2+^ (with lower binding energy) and Fe^3+^ (with higher binding energy), respectively. The calculated content ratio of Fe^2+^ to Fe^3+^ is 22.5%, 13.8% and 27.4% for S1–S3 (Table 1), respectively. The presence of Fe^2+^ in the spinel structure of ZnFe_2_O_4_ will break the balance of charge distribution, leading to the charge redistribution and dipoles formation. S3 has a larger Fe^2 +^ /Fe^3+^ ratio, which definitely introduces more dipoles into the absorber, thereby promoting the EM wave dissipate ability.

**Figure 5:**
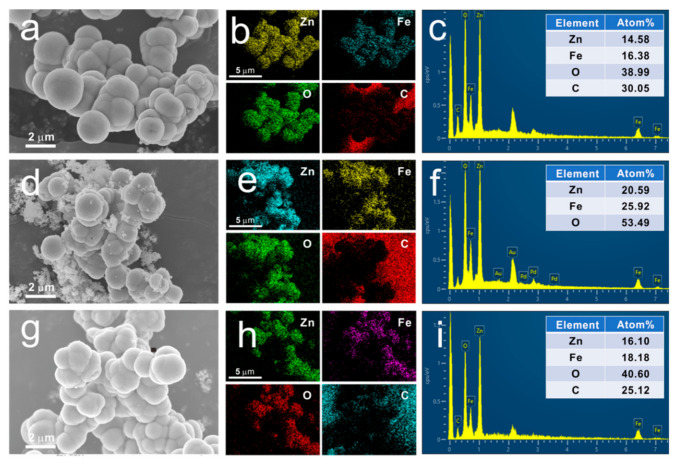
SEM images of S1 (**a**), S2 (**d**) and S3 (**g**); EDS mapping of S1 (**b**), S2 (**e**) and S3 (**h**); EDS spectra of S1 (**c**), S2 (**f**) and S3 (**i**).

In order to investigate the morphologies of the samples, SEM images of S1–S3 were recorded and shown in Figure 5. As can be seen in Figure 5a, the as-prepared sample (namely S1) has a spherical microstructure with a diameter of about 1–2 μm. These microspheres with rough surfaces are formed by numerous crystallites with scales ranging from a few nanometers to tens of nanometers. When the as-prepared samples were annealed in air, the carbon could be removed and hollow spherical structures were obtained (Figure 5d), while solid microspheres were formed after annealing in N_2_ (Figure 5g). To study the composition of the samples at the atomic scale, EDS mapping and EDS spectra of S1–S3 were measured and presented in Figure 5b,e,h and 5c,f,i, respectively. The EDS mapping images show that Zn, Fe, and O atoms are uniformly distributed in all samples. In addition, the signal of C atom could be clearly observed for S1 and S3, while it is hardly detected for S2 due to the removal of carbon after air-annealing. 

The atomic content of each element was further calculated from the EDS spectra and the results show that the atomic content of each element in S1 is 14.58%, 16.38%, 38.99% and 30.05% for Zn, Fe, O and C, respectively. The atomic ratio of Zn to Fe is about 0.89, which is higher than the stoichiometric ratio of Zn to Fe in ZnFe_2_O_4_ (which is 0.5). The previous analysis of XRD, Raman and PL results shows that S1 is composited of ZnFe_2_O_4_, ZnO, and carbon. Combined with the results of EDS spectra, the content ratio of ZnO to ZnFe_2_O_4_ in S1 is calculated to be ~0.78, which is ~0.59 and ~0.77 for S2 and S3 (Table 1), respectively. Larger values close to 1 suggests that more ZnFe_2_O_4_/ZnO interfaces may exist in S1 and S3 than in S2, and the presence of carbon also enriches the types of interfaces in S1and S3 by forming ZnFe_2_O_4_/C and ZnO/C interfaces. Thus, it can be predicted that stronger interfacial polarizations and relaxations may occur in S1 and S3 under alternating electromagnetic field.

**Figure 6:**
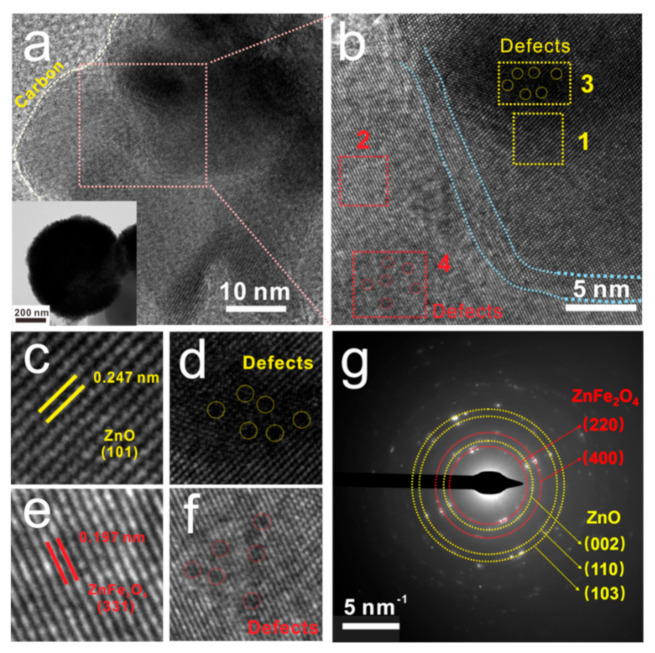
TEM pattern (insert in (**a**)), HR-TEM images (**a**–**f**) and SAED patterns (**g**) of S2.

The microstructure, interfaces and defects of the samples were further characterized by TEM images. As shown in the insert of Figure 6a, S3 has a solid spherical microstructure, which is consistent with the results observed from SEM images. The HR-TEM image (Figure 6a) shows the microsphere is composited by graphite carbon and crystal grains with a clear interface between them, as marked by the white dashed line. The enlarged HR-TEM image (Figure 6b) further reveals that the grains contain two different crystals and the interface between them (marked by the light blue dashed line) can be clearly observed. The interplanar spacing of the crystals is measured to be 0.247 nm (Figure 6c) and 0.197 nm (Figure 6e), which corresponds well to the (101) planes of ZnO and (331) plane of ZnFe_2_O_4_, respectively. The SAED pattern further confirms the coexistence of ZnO and ZnFe_2_O_4_ in S3, with diffraction rings related to typical interplanar spacing of ZnO and ZnFe_2_O_4_ appearing in the pattern. In addition to the interfaces, lattice defects also widely exist both in ZnO and ZnFe_2_O_4_ (Figure 6b,d,f). These defects disrupt the periodic structure of the crystals and alter the electric field as well as the electron transport paths, thereby hindering the movement of free electrons and forming accumulated charges. When exposed to an alternating electromagnetic field, the accumulated charge undergoes periodic polarization and relaxation, which dissipates the energy of the EM waves. 

**Figure 7:**
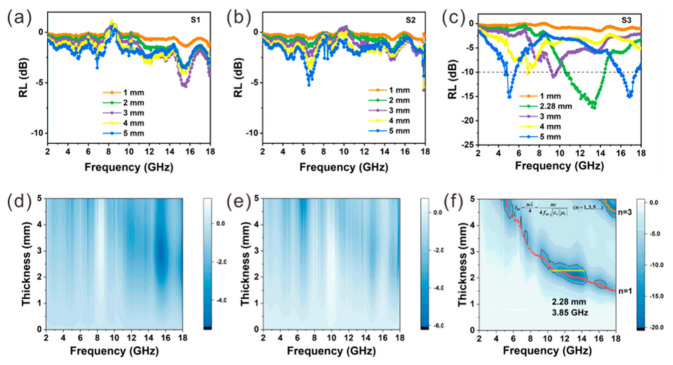
The 1D and 2D diagrams of the RL value changing with frequency and thickness: (**a**,**d**) S1, (**b**,**e**) S2 and (**c**,**f**) S3.

To evaluate the EMW absorption capability of the samples, the reflection loss (RL) values were calculated by using the following equations [44]:

(1) $$RL(dB)=20\log\left|\frac{Z_{in}-Z_{0}}{Z_{in}+Z_{0}}\right|$$

(2) $$Z_{in}=Z_{0}\sqrt{\frac{\mu_{r}}{\varepsilon_{r}}}\tanh\left|j(\frac{2\pi fd}{c})\sqrt{\varepsilon_{r}\mu_{r}}\right|$$

where *Z_in_* and *Z*_0_ represent the input impedance of the absorber and air, $\mu_{r}$ and $\varepsilon_{r}$ are complex permeability and permittivity, *f* is the frequency of the incident EMW, *d* is the thickness of the absorber, and *c* is the speed of light in vacuum. Figure 7 shows the *RL* value of the samples as a function of frequency and thickness. A minimum reflection loss (*RL_min_*) value of −5.4 dB is obtained for S1 at 15.6 GHz with a thickness of 3 mm (Figure 7a,d), while the *RL_min_* value for S2 is −5.2 dB at 6.6 GHz with a thickness of 5 mm (Figure 7b,e). For S1 and S2, the *RL_min_* was obtained at different frequencies that do not shift with thickness, which suggests that different EMW absorption mechanisms may exist for S1 and S2, respectively. Compared with S1 and S2, S3 exhibits significantly enhanced EMW absorption performance with a wide effective absorption bandwidth (EAB, expressed in *f*_e_) of 3.85 GHz at 2.28 nm as well as small *RL_min_* value of -17.4 dB at 13.44 GHz (Figure 7c,f). 

Moreover, the *RL_min_* shifts to lower frequencies with the increase in thickness, which can be explained by quarter-wavelength theory based on the following equation [45]:

(3) $$t_{m}=\frac{n\lambda }{4}=\frac{nc}{4f_{m}\sqrt{\left|\varepsilon_{r}\right|\left|\mu_{r}\right|}}\;(n=1,3,5\ldots )$$

where *t_m_* is the matching thickness, and *λ* and *f_m_* represent the wavelength and the frequency of the EMW, respectively. The calculated data were plotted in Figure 7f, with red and orange curves obtained when *n* equals π1 and 3, respectively. It is clear that the frequencies of *RL_min_* can be well fitted to the $t_{m}∼\lambda /4$ curve, suggesting that the shift in *RL_min_* originates from the destructive interference of incident waves and reflected waves, which dissipate each other at the air–absorber interface due to the phase difference of π, thereby improving the EM wave attenuation capacity.

**Figure 8:**
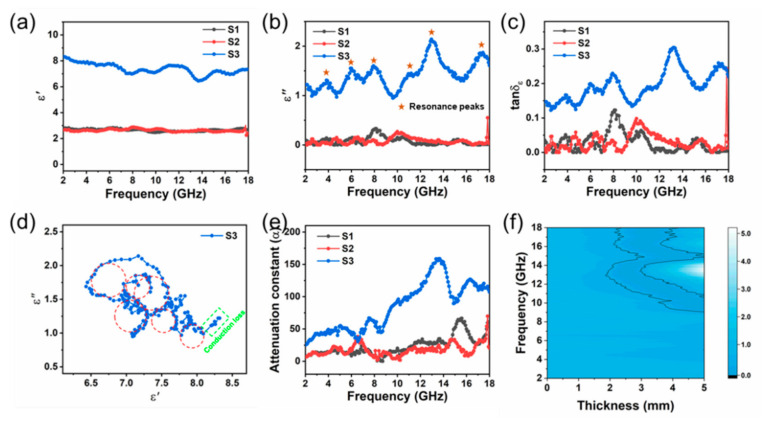
Frequency dependence of (**a**) *ε*′ (**b**) *ε*″ and (**c**) dielectric loss tangents of S1–S3; (**d**) Cole–Cole semicircles of S3; (**e**) the attenuation constant of S1–S3; (**f**) the calculated delta value maps of S3. (Black line, |Δ| = 0.4).

In order to clarify the mechanism of the enhanced EMW absorption capability of S3, the complex permeability ($\mu_{r}=\mu^{\prime }-j\mu^{\prime \prime }$) and permittivity ($\varepsilon_{r}=\varepsilon^{\prime }-j\varepsilon^{\prime \prime }$) of all samples were plotted in Figure S1a,b and Figure 8a,b, respectively. For all samples, the values of $\mu^{\prime }$ fluctuate around 1 and the $\mu^{\prime \prime }$ values as well as the magnetic loss tangent ($\tan\delta_{\mu }=\mu^{\prime \prime }/\mu^{\prime }$) get close to 0 in 2–18 GHz, implying their poor magnetic loss capability. In general, the magnetic loss in gigahertz range is mainly derived from natural resonance and the eddy current effect, the contribution of which can be judged by the following equation [46]:

(4) $$C_{0}=\mu^{\prime \prime }{\left(\mu^{\prime }\right)}^{-2}f^{-1}=2\pi \mu_{0}d^{2}\sigma$$

where *f* is the frequency of incident EM wave, and $\mu_{0}$ and σ are the vacuum permeability and electric conductivity, respectively. If magnetic loss is dominated by the eddy current, the left side of the formula will be a constant. As displayed in Figure S1d, the values of all samples are barely changed in 8–18 GHz, suggesting the predominant contribution of eddy current in this frequency range. Several peaks appear in the low frequency of 2–8 GHz, which correspond to natural resonances in the samples. Since the magnetic loss tangent has similar values for all samples, the magnetic loss will show comparable contribution to the EM wave dissipation. Thus, dielectric loss needs to be taken into consideration for the enhanced dissipation ability of S3.

As shown in Figure 8b,c, S3 has much larger $\varepsilon^{\prime \prime }$ value and dielectric loss tangent ($\tan\delta_{\varepsilon }=\varepsilon^{\prime \prime }/\varepsilon^{\prime }$) than S1 and S2 over the entire frequency range, indicating that S3 has the best dielectric loss capacity among all three samples, which may be the main reason for its enhanced EMW absorption performance. Generally, dielectric loss in gigahertz frequency region originates from polarization relaxation dominated by dipole polarization and interfacial polarization, both of which can generate Debye-like relaxations under an alternating EM field. These Debye relaxation processes cause the resonance peaks in the $\varepsilon^{\prime \prime }$ and $\tan\delta_{\varepsilon }$ spectra of S3, as shown in Figure 8b,c. The Debye relaxations can be further characterized by the Cole–Cole semicircles, as described by the following equation [25]:

(5) $${\left(\varepsilon^{\prime }-\frac{\varepsilon_{s}+\varepsilon_{\infty }}{2}\right)}^{2}+{\left(\varepsilon^{\prime \prime }\right)}^{2}={\left(\frac{\varepsilon_{s}-\varepsilon_{\infty }}{2}\right)}^{2}$$

where $\varepsilon_{s}$ and $\varepsilon_{\infty }$ are the static and infinite frequency dielectric constants, respectively. As shown in Figure 8d, multiple Cole–Cole semicircles exist for S3, and their frequencies correspond well with those of the peaks in Figure 8b,c. In terms of S1 and S2, the resonance peaks can also be observed, but with much weaker intensities. The indistinguishable semicircles in their Cole–Cole curves (Figure S2) validate the poor dielectric loss ability of S1 and S2.

As mentioned above, dielectric constants and dielectric loss are closely related to lattice defects and interfaces in materials. According to PL, XPS and TEM results, multiple lattice defects (such as V_O_, V_Zn_ and Zn_i_ in ZnO, and deep-level defects in ZnFe_2_O_4_) exist in our samples, which can serve as charge accumulation centers and generate dipoles with varied dipole moment. These defect-induced dipoles will periodically polarize and relax under alternating electromagnetic field, resulting in dielectric loss and EM energy dissipation. Table 1 lists the content of various types of lattice defects in S1–S3. Compared with S1 and S2, S3 has more Zn-related defects (V_Zn_ and Zn_i_) and a larger Fe^2+^/Fe^3+^ ratio as well as high oxygen vacancy concentration and high disorder degree in graphite carbon, indicating that stronger dipole polarizations will occur in S3, which leads to significant increase in the permittivity ($\varepsilon^{\prime \prime }$) and the dielectric loss ($\tan\delta_{\varepsilon }$), thereby promoting the EM wave dissipation ability of S3.

In addition to defects-induced dipoles, space charges accumulated at the boundaries between various components in the material may also contribute to the polarization and dielectric loss. Based on the classic Maxwell–Wagner model, the $\varepsilon^{\prime \prime }-\omega$ characteristics for interfacial polarization can by described by the following equation [47]:

(6) $$\varepsilon^{\prime \prime }=\frac{\omega \tau \left(\varepsilon_{s}-\varepsilon_{\infty }\right)}{1+\omega^{2}\tau^{2}}+\frac{\sigma }{\omega \varepsilon_{0}}$$

where ω, τ and σ are the angular frequency, polarization relaxation time and electrical conductivity, respectively. The first term of the equation is the same as the Debye relaxation, and at higher frequencies, the relaxation for interfacial polarization is indistinguishable from dipolar relaxation, suggesting that the interfacial polarization, such as defects-induced dipole polarization, will also cause dielectric loss and EM energy dissipation. As analyzed above, ZnFe_2_O_4_/ZnO interfaces widely exist in all three samples, and the ratio of ZnO to ZnFe_2_O_4_ is ~0.78, ~0.59 and ~0.77 for S1, S2 and S3, respectively. The larger value approaching 1 indicates that there will be more ZnFe_2_O_4_/ZnO interfaces in S1 and S3. In addition, due to the presence of amorphous or graphite carbon, ZnFe_2_O_4_/C and ZnO/C interfaces were also formed in S1 and S3 (as shown in Figure 6b), which enriches the variety and quantity of interfaces. Compared with S3, the excessive content of amorphous carbon in S1 will reduce the total number of interfaces to a certain extent. Thus, S3 with the appropriate content of each component possesses the largest number of interfaces, leading to the strongest interfacial polarization under alternating EM waves.

Apart from the polarization and relaxation, the conductivity is another key factor affecting the permittivity of a material by causing conduction loss, as expressed by the second term of Equation (6), which makes an increasing contribution to the dielectric loss as the frequency becomes smaller and the conductivity become larger. Generally, D-glucose is carbonized to amorphous carbon after the hydrothermal treatment, and it can be further converted into graphitic carbon with better conductivity by annealed in nitrogen or argon [48]. It can be predicted that the presence of large amount of conductively graphite carbon in S3 will provide electron pathways and ensure the free charges flow through the materials forming current, thereby resulting in conduction loss and energy dissipation. As shown in Figure 8d, an approximately linear tail exists at the right end of curve (low frequency range), confirming the presence of conduction loss in S3. Meanwhile, due to the amorphous carbon with low conductivity in S1 and the absence of carbon in S2, the conduction loss is hardly detected in these two samples.

As analyzed above, it can be concluded that under certain conditions, the introduction of dielectric loss materials (ZnO and C) can effectively enhance the EMW absorption performance of ZnFe_2_O_4_. First, the content of dielectric loss materials should be appropriate, since insufficient or excess dielectric loss materials will cause a negative effect, such as impedance mismatch and reduce of interfaces. Among the three samples, S3 has the optimize content for each component. Second, abundant lattice defects will benefit the EMW absorption, which can cause defect polarization and dipole polarization under alternating EM field. Large number of lattice defects formed in ZnO and ZnFe_2_O_4_ crystals after annealing in N_2_, resulting in the high content of defects in S3. Third, good conductivity is also important. Compared with S1 and S2, S3 has better conductivity due to the presence of graphite carbon. Therefore, S3 shows better EMW absorption performance than S1 and S2, due to the proper composite content, abundant lattice defects and good conductivity.

The total EM wave attenuate ability of the absorbers was further evaluated by attenuation constant (*α*), which can be calculated by the following formula [49,50]:

(7) $$\alpha =\frac{\sqrt{2}\pi f}{c}\times \sqrt{(\mu^{\prime \prime }\varepsilon^{\prime \prime }-\mu^{\prime }\varepsilon^{\prime })+\sqrt{{\left(\mu^{\prime \prime }\varepsilon^{\prime \prime }-\mu^{\prime }\varepsilon^{\prime }\right)}^{2}+{\left(\mu^{\prime }\varepsilon^{\prime \prime }+\mu^{\prime \prime }\varepsilon^{\prime }\right)}^{2}}}$$

The α value of S1–S3 shows similar fluctuating upward trend in 2–18 GHz, while S3 has larger *α* values than S1 and S2 in the entire range (Figure 8e), which indicates that it has the best attenuation ability among these samples. The *α* plot of S3 has a similar shape with that of its dielectric loss tangent, confirming that excellent attenuation performance of S3 is dominated by dielectric loss caused by defects-induced dipole polarization and interfacial polarization, as well as the conduction loss.

In addition to the attenuation capacity, impedance matching also plays an important role in determining the absorption behavior of absorbers, which can be evaluated by the following formula [51,52]:

(8) $$\left|\Delta \left|=\right|\sinh^{2}(Kfd)-M\right|$$

(9) $$K=\frac{4\pi \sqrt{\mu^{\prime }\varepsilon^{\prime }}\times \sin\left(\frac{\delta_{e}+\delta_{m}}{2}\right)}{c\times \cos\delta_{e}\times \cos\delta_{m}}$$

(10) $$M=\frac{4\mu^{\prime }\cos\delta_{e}\times \varepsilon^{\prime }\times \cos\delta_{m}}{{\left(\mu^{\prime }\cos\delta_{e}-\varepsilon^{\prime }\cos\delta_{m}\right)}^{2}+{\left[\tan\left(\frac{\delta_{m}}{2}-\frac{\delta_{e}}{2}\right)\right]}^{2}{\left(\mu^{\prime }\cos\delta_{e}+\varepsilon^{\prime }\cos\delta_{m}\right)}^{2}}$$

The calculated |Δ| value maps of all samples were displayed in Figure S3 and Figure 8f and the area of $\left|\Delta \right|\leq 0.4$ is commonly used to evaluate the impedance matching. Obviously, S3 exhibits best optimal impedance matching with the largest area of $\left|\Delta \right|\leq 0.4$, which facilitates the entry of incident EM waves into interior of the absorbers, thereby improving the EMW absorption performance of S3.

**Figure 9:**
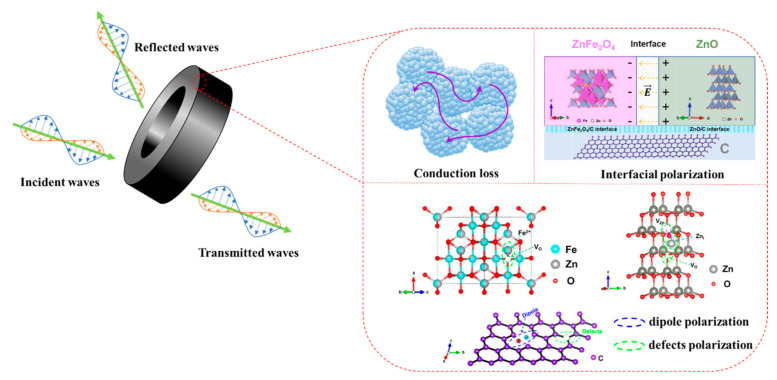
Schematic diagram of the microwave absorption mechanism in ZnFe_2_O_4_/ZnO/C composites.

According to the above analysis, the excellent EM wave dissipation ability of S3 originates from multiple loss processes, as depicted in Figure 9. First, the well-matched impedance ensures that most of the incident EM wave effectively enter the absorber. Second, the quarter-wavelength interference causes cancellation between the incident and reflected EM waves, enhancing the attenuation performance. Third, due to the presence of conductively graphite carbon in the sample, conduction loss will occur when the incident EM wave enters the absorbers, which helps to dissipate the EM wave energy. Last, dielectric loss caused by interfacial polarization, defects polarization and dipole polarization further strengthen the EMW consumption ability, resulting in a *RL_min_* value of −17.4 dB and an effective absorption bandwidth of 3.85 GHz at 2.28 mm for S3.

## 4. Conclusions

In this study, ZnFe_2_O_4_/ZnO/C composite with excellent EMW absorption performance was synthesized by the hydrothermal method and post-annealing process in different atmospheres. Defects and interfaces were investigated by various characterizations, including Raman, PL spectroscopy, XPS and TEM. The results show that the N_2_-annealed sample (S3) has more Zn-related defects (V_Zn_ and Zn_i_) and a larger Fe^2+^/Fe^3+^ ratio as well as high oxygen vacancy concentration and high disorder degree in graphite carbon. Due to the presence of conductive graphite carbon and the optimized composition, S3 also possesses the most interfaces and best impedance matching, as well as significant conduction loss under an alternating EM field. The suitable impedance matching, significant conduction loss and strong dielectric loss (interfacial polarization, diploe polarization and defect polarization) dominated by lattice defects and interfaces result in an excellent EMW dissipation performance for S3, with a *RL_min_* value of −17.4 dB and an *f_e_* of 3.85 GHz at 2.28 mm. This study provides a view into the relationship between defects, interfaces, EM parameters and EMW absorption ability, and it shows that the EMW dissipation ability of the absorbers can be effectively promoted by controlling defects and interfaces via the post-annealing treatment.

## Figures and Tables

**Table 1:** Components, defects, permittivity, dielectric loss and EMW absorption performance of S1–S3.

Sample	Components & Interfaces	Defects				Permittivity		tan *δ_ε_*	*RL_min_* & *f_e_*
	ZnFe_2_O_4_:ZnO:C	I_D_/I_G_	Zn_i_&V_Zn_	V_O_	Fe^2+^/Fe^3+^	*ε*′	*ε*″		
S1	1:0.78:3.67	0.89	0.89	45.30%	22.50%	~3.0	~0–~0.35	~0–~0.12	−5.4 dB, -
S2	1:0.59:0	-	0.91	16.10%	13.80%	~3.0	~0–~0.30	~0–~0.10	−5.2 dB, -
S3	1:0.77:2.76	0.91	1.18	35.10%	27.40%	~6.8–~8.3	~1.0–~2.2	~0.12–~0.3	−17.4 dB, 3.85 GHz

## Data Availability

Not applicable.

## Supplementary Materials

The following supporting information can be downloaded at: https://www.mdpi.com/article/10.3390/nano12162871/s1, Figure S1. Frequency dependence of *μ*′ (a), *μ*″ (b), magnetic loss tangents (c) and C_0_ values (d) of S1–S3. Figure S2. Cole–Cole plots of S1 (a) and S2 (b). Figure S3. The calculated delta value maps of S1 (a) and S2 (b).
