# Supplementary material for: Defect- and Interface-Induced Dielectric Loss in ZnFe2O4/ZnO/C Electromagnetic Wave Absorber
Source: Nanomaterials (Basel). 2022 Aug 20;12(16):2871. doi: 10.3390/nano12162871 (PMC9414697; doi:10.3390/nano12162871)
Supplement: Supplementary file 1 [file nanomaterials-12-02871-s001.zip › nanomaterials-1853042-supplementary.pdf]

Defect- and Interface-Induced Dielectric Loss in $ZnFe_2O_4$/ZnO/C Electromagnetic Wave Absorber

Hao Shen [1], Zhen Wang [1], Chun Wang [2], Pengfei Zou [1], Zhaoyang Hou [1], Chunlong Xu [1,*] and Hongjing Wu [3,*]

[1] Department of Applied Physics, School of Science, Chang'an University, Xi'an 710064, China
[2] School of Science, Xi'an Shiyou University, Xi'an 710064, China
[3] MOE Key Laboratory of Material Physics and Chemistry Under Extraordinary Conditions, School of Physical Science and Technology, Northwestern Polytechnical University, Xi'an 710072, China
* Correspondence: chunlongxu@chd.edu.cn (C.X.); wuhongjing@nwpu.edu.cn (H.W.)

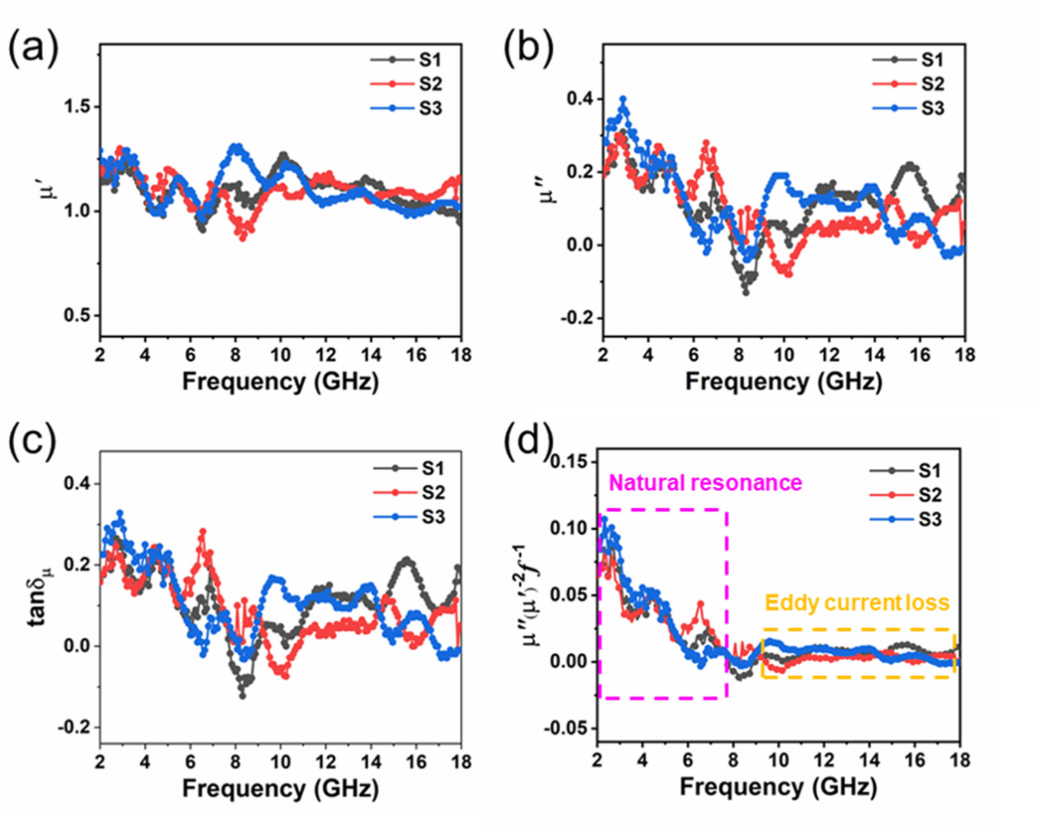

Figure S1. Frequency dependence of μ' (**a**), μ'' (**b**), magnetic loss tangents (**c**) and C_0 values (**d**) of S1–S3.

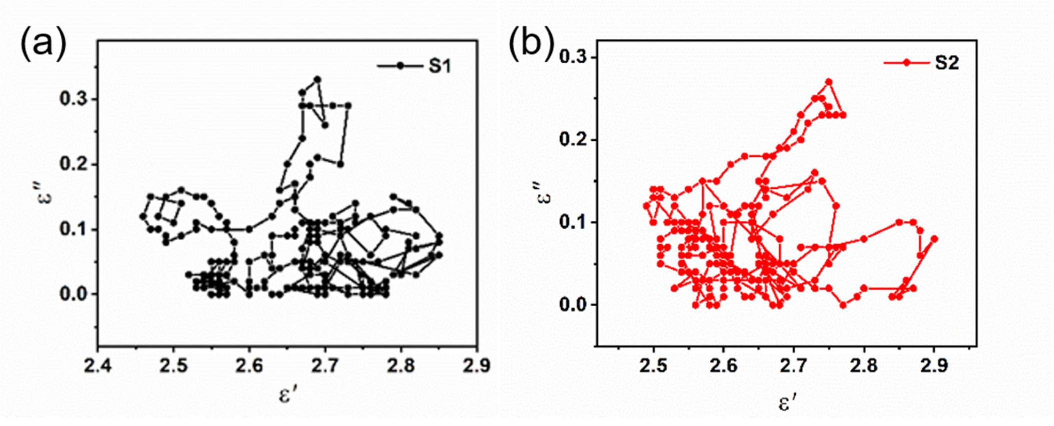

Figure S2. Cole-Cole plots of S1 (a) and S2 (b).

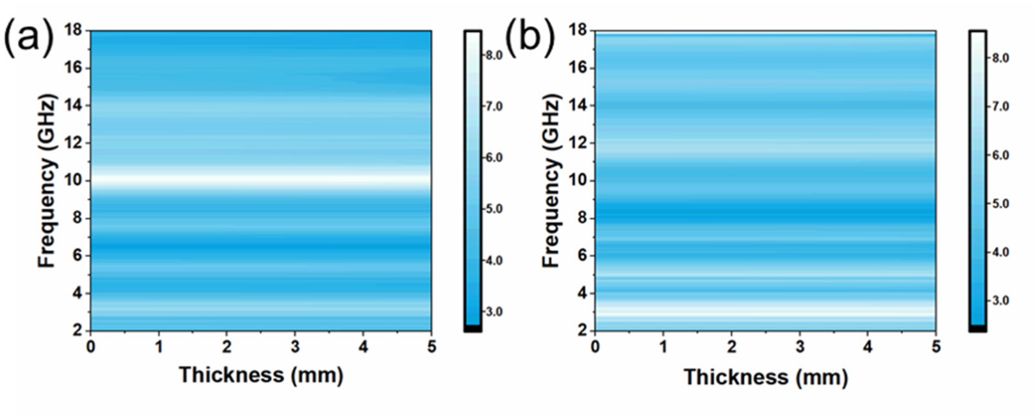

Figure S3. The calculated delta value maps of S1 (a) and S2 (b).
